# Inequity in Schoolchildren’s Access to Oral Health Services in Romania: Implications for Public Oral Health Policies

**DOI:** 10.3390/healthcare14030415

**Published:** 2026-02-06

**Authors:** Anca-Cristina Perpelea, Ruxandra Sfeatcu, Silviu-Mirel Pițuru, Florentina Ligia Furtunescu

**Affiliations:** 1 Department of Organization, Professional Legislation and Management of the Dental Office, Faculty of Dentistry, “Carol Davila” University of Medicine and Pharmacy, 17-23 Plevnei Street, 020021 Bucharest, Romania; 2 Oral Health and Community Dentistry Department, Faculty of Dentistry, “Carol Davila” University of Medicine and Pharmacy, 17-21 Calea Plevnei Street, 010221 Bucharest, Romania; ruxandra.sfeatcu@umfcd.ro; 3 Discipline of Public Health and Management, Faculty of Medicine, “Carol Davila” University of Medicine and Pharmacy, 050474 Bucharest, Romania

**Keywords:** oral public health, health promotion, oral health services, vulnerable population, health equity, oral health determinants, oral health policies

## Abstract

**Background/Objectives**: In Romania, the oral health status of schoolchildren is insufficiently documented at the national level. This is due to the lack of systematic oral health reporting and fragmented access to dental care services. **Methods**: In this context, the main factors influencing schoolchildren’s access to dental services were identified through a triangulation approach that integrated documentary research, routine data on health service coverage, and oral health indicators collected through a survey conducted on 3843 schoolchildren. **Results**: The results highlight a multitude of interdependent factors that shape children’s access to dental care, namely policy-level constraints, dental workforce distribution and individual-level determinants—and provide insight into how public health policies can be adapted to more effectively meet the needs of this group. **Conclusions**: Despite legislative provisions on children’s oral health care in Romania, territorial disparities persist, and the use of preventive services remains low. These findings underscore the need for national- and county-level public health policies to improve access, promote preventive dental check-ups, strengthen health literacy, and develop targeted programs specifically dedicated to school-age children.

## 1. Introduction

Since 1990, oral diseases have been the most common health issue worldwide [1], with incidence remaining high over the past 30 years [2]. The Global Burden of Disease Study reports that approximately half of the population was affected by oral conditions [2,3], a prevalence that exceeds that of any other non-communicable condition [4]. It is also well established that oral conditions can be associated with general health conditions [5,6,7,8]. The World Health Organization aims to reduce the burden of oral conditions and has developed an action plan that focuses on priority intervention areas [9]. Oral health is a multifactorial public health issue influenced by structural determinants (such as access to services and economic policies) [10]. The limited availability of dental services creates inequalities and inequities in the provision of care [11,12,13,14].

Access to dental care is described as a complex framework encompassing multifactorial elements and various determinants [15,16], such as the accessibility and availability of oral health systems, general and oral health status, and the legislative framework [16,17,18,19,20,21,22,23,24,25]. In addition, subjective perception of oral health and normative needs for dental care shape the use of dental services [26]. Assessing dental service use is essential for identifying inequalities, understanding their determinants, and monitoring the unmet needs. Self-reported unmet need for dental examinations by sex, age, main stated reason, and income quintile [27], and the linkage between the dentist coverage rate and children’s oral health/attendance has not been analyzed in Romania.

Accessibility, availability, and affordability of dental care: In Romania, public and private sectors deliver dental services [28]. The health system operates on a social health insurance model. According to national legislation, children under 18 are entitled to free medical services, and the framework agreement stipulates that each child is entitled to two free consultations per year. Healthcare services are provided across 41 counties and in Bucharest, in accordance with regulations set by central authorities. Within the social health insurance system, district health insurance funds purchase services from local providers [29]. However, unlike hospital-based medical care, dental care is funded by public resources only to a limited extent. It is mainly financed through patients’ out-of-pocket payments.

Oral health in Romanian children is fragmentarily known and shows poor or very poor indicators, even though the health system provides, at least according to legal stipulations, free dental care for all children.

This article aims to explore the association between children’s oral health, access to oral health services, and utilization of these services. The null hypothesis assumed that there is no significant association between children’s oral health status and their access to dental offices or utilization of oral health services among Romanian schoolchildren.

The objectives of the study were to examine potential inequalities in access to and use of oral health services, and their association with oral health outcomes. The findings may help inform national oral health policy and SDG/WHO oral health strategies by providing the first integrated picture of access inequities in Romanian schoolchildren.

## 2. Materials and Methods

To achieve this objective, we performed a mixed-method study, using triangulation of desk research for legislation referring to oral health access policies in Romania, survey-based data on oral health status and service utilization, and data from the routine information system regarding coverage of oral health services. The first stage consisted of conducting desk research to evaluate the regulatory and institutional framework that defines Romania’s oral health system. Thus, the legislative provisions of Law No. 95/2006 on health reform and the Framework Contract for health services provision issued by the National Health Insurance House have been analyzed, following the access policies to oral health services.

The second stage of the study was conducted between March and September 2025 and consisted of an in-depth analysis of data from national routine systems. The data were extracted from the National Institute of Statistics—Tempo Online platform [30]. Data were collected on the number of dentists registered in Romania in 2023, and on the population size in the same year, by county of residence. Subsequently, the dentist coverage rate was calculated by county and by the type of sector in which dentists practice (public/private). The coverage rate was calculated as the number of dentists divided by the county’s total population, multiplied by 100,000.

The final stage consisted of an analysis of the determinants of children’s oral health status. This component was based on the World Health Organization framework [31], provided in 2013. The survey used a standardized, validated questionnaire and a clinical medical record. The activities were conducted between 2022 and 2023, in accordance with the methodology implemented and regulated at the national level [32]. Data from 3843 schoolchildren were analyzed. The study was conducted in counties with school dental clinics and dentists (35 counties and Bucharest). Its description was provided in previously published articles [23,24,25]. This approach aimed to identify access-related elements from an individual perspective.

SPSS Statistics, version 29.0, was used to calculate central tendency and dispersion measures for all four caries indices (dmfs, dmft, DMFS, DMFT), including means and standard deviations. Mean values were compared, and dentist coverage per 100,000 inhabitants by county was calculated using the resident population as of 1 January 2023. Spearman bivariate correlations were computed between the four indices and dentist coverage.

### Ethical Consideration

The Research Ethics Committee of the “Carol Davila” University of Medicine and Pharmacy in Bucharest approved the study (protocol number 36987/29 November 2022). Written informed consent was obtained from the legal representatives of all participating schoolchildren prior to data collection.

## 3. Results

Desk research—Availability of dental services in Romania: The Romanian healthcare system is mainly financed through social insurance, and all children are insured without paying contributions [33]. Dental services are available nationwide; however, they are primarily concentrated in urban areas, and providers can be either public or private. Public providers operate under contracts with the National Health Insurance House (NHIH) or receive government funding (such as school dental offices). Private providers may enter into service contracts with health insurance funds; however, their interest in doing so is usually low because the Health Insurance Fund caps contracts at 6000 RON per month (approximately 1200 euros) in urban areas. In rural areas, the contracting limit is higher—9000 RON per month (approximately 1800 euros)—but this still does not provide enough incentive to attract dentists [29].

Legislative provisions that stipulate complex bureaucratic procedures for funding justification, the low level of financial allocation, and the manner in which this funding must be justified are factors that substantially reduce private providers’ interest in contracting with the NHIH. As a result, parents seeking dental services for their children turn to private providers outside the social insurance system. They must bear the full cost of medical services, which represents a significant barrier to access to dental care.

Additionally, the NHIH funds allocated to oral health account for less than 0.5% of total public health expenditures. According to NHIH data, nationwide in 2023, a total of 3561 contracts were concluded with dental practitioners, and 1,839,365 therapeutic procedures were reimbursed (without disaggregation by age group). Data indicate that, in the same year, approximately one therapeutic act was reimbursed for every 10 inhabitants in Romania [34].

Data from national routine information systems show that, in 2023, Romania had 21,200 practicing dentists. No distinction was made between licensed and practicing doctors; 3771 worked in the public sector and 17,429 in the private sector. Of the total number of dentists, 18,905 practice in urban areas, whereas only 2295 practice in rural areas.

In line with the relatively large proportion of the population living in rural areas (45%), considerable disparities are evident in the territorial distribution of dentists. This distribution gap disadvantages rural communities. This is a factor that leads to notable inequalities, including inequalities in children’s access to dental care services.

**Figure 1:**
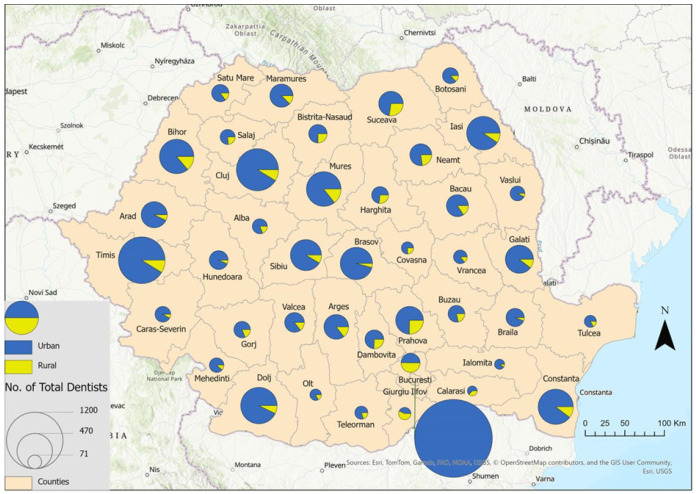
Number of dentists in Romania, in 2023. Data source: National Institute of Statistics—Tempo Online platform.

The distribution of dentists by county and by practice setting is illustrated in Figure 1. Differences in the distribution of dentists across counties are evident. The data highlight dentists’ preference for practicing in urban areas and large university cities (e.g., Bucharest, Timis, Cluj, Constanta). In the counties Calarași, Ialomita, Olt and Tulcea, there are a small number of dentists—71, 76, 97 and 108.

**Figure 2:**
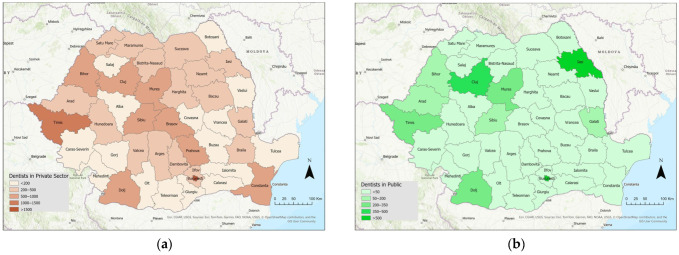
The number of dentists practicing in the (**a**) private health system; (**b**) public health system. Data source: National Institute of Statistics—Tempo Online platform.

Figure 2a,b illustrates the distribution of dentists practicing in the public and private sectors. The data show a discrepancy between the number of dentists practicing in the public system and those who are practicing in the private system. It is evident that, in the majority of counties, the number of dentists practicing in the public system does not exceed 50. The number of dentists working in the private sector exceeds 200 in most counties nationwide. This 4-to-1 private–public ratio may represent a barrier to schoolchildren’s access to oral health services.

**Figure 3:**
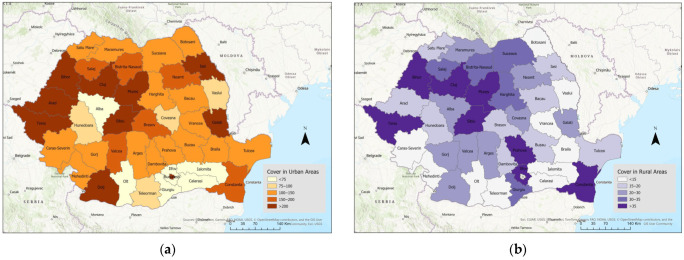
Dentist coverage rate in the (**a**) urban areas, (**b**) rural areas; county-level distribution. Data source: National Institute of Statistics—Tempo Online platform.

Figure 3a,b illustrates the dentist coverage rate in urban and rural areas. The disparities in dentist coverage rates across counties are evident. There are a few counties with a high coverage rate in both rural and urban areas (Timis, Bihor, Cluj, Mures, Sibiu, and Constanta). In urban areas, some counties reach coverage levels of 200 or more (Timis, Dolj, Iasi, etc.), while other regions report rates approximately three times lower (e.g., Alba, Olt, Ialomita, Calarasi). In rural areas, the coverage rate exceeds 35 in 8 counties, and is less than 15 in 10 counties. Consequently, inequalities in access to dental services are observed, including for children.

**Figure 4:**
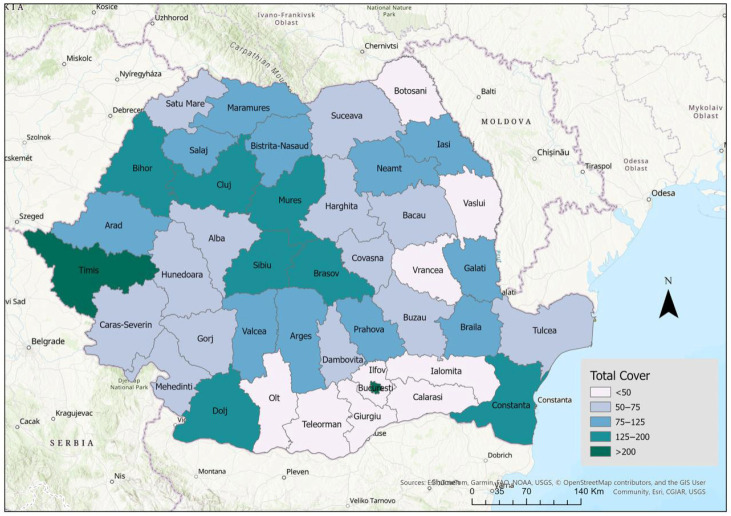
Dentist coverage rate, county-level distribution. Data source: National Institute of Statistics—Tempo Online platform.

The total dentist coverage rate (urban and rural areas), at the county level, is shown in Figure 4. In Romania, major discrepancies exist between counties, suggesting the need for county-specific action plans to reduce territorial distribution inequalities.

Data on oral health indicators collected through a survey—For the analyzed sample, according to the previously published data, mean dmfs ± SD = 6.07 ± 7.4, mean dmft ± SD = 3.11 ± 2.91, mean DMFS ± SD = 2.48 ± 4.76, mean DMFT ± SD = 1.77 ± 2.86 [24] (Table 1).

Spearman’s rank correlation analysis revealed statistically significant associations among the mean caries indices, indicating an association between the severity of dental caries in the primary and permanent dentition and suggesting that a higher tooth decay burden at early ages may be an important predictor of caries development in the permanent dentition. Regarding the correlation between patterns of dental visits among Romanian schoolchildren reported in the survey and dentist coverage analyzed using data from the national routine system, an inverse association was observed between low dental attendance (fewer than two dental visits) and dentist coverage (negative correlation coefficient, r = −0.172). Even though the correlations are modest and did not reach statistical significance (*p* = 0.316 > 0.05), the observed tendency provides significant descriptive information. This result indicates that beyond dentist coverage rates, additional factors can influence schoolchildren’s access to oral health services.

## 4. Discussion

### 4.1. Interpretation of Results and Policy Implications

Based on the results presented above, a wide range of factors can influence Romanian schoolchildren’s access to dental services. The modest correlations observed among the factors indicate that the presence of dental services alone cannot fully explain the variations in children’s oral health outcomes. Therefore, in order to analyze the factor “access to dental services” and to outline guiding principles for oral health policies in Romania, it is necessary to consider three complementary perspectives:

1. Policy-level constraints: limited reimbursement caps, absence of a mechanism for workforce distribution in relation to the territorial distribution of the population.
2. Workforce distribution and oral health services infrastructure. There are major disparities in the distribution of dentists. Oral health services for children are insufficient in some regions of the country, unevenly distributed, and inadequately utilized. To address territorial-level inequalities in access, regionally differentiated, targeted measures are needed.
3. Individual-level determinants. In line with the limited evidence that shows a fragmented picture of Romanian schoolchildren’s oral health [35], understanding the pattern of dental service use is very important. Service utilization and oral health status are influenced by a comprehensive set of factors, including adults’ educational level and health literacy. Therefore, Romania must develop oral health policies that ensure all children know the oral health determinants. Awareness can be raised through oral health policies that support educational programs and interventions. This can be implemented in public or private services, as well as within educational institutions.

### 4.2. Recommendation

Given the Romanian oral health system’s specific financing and organizational characteristics, we propose these interventions:

1. Counties with low dentist coverage rates (Olt, Teleorman, Călărași, Ialomița, etc.) could benefit from county-specific actions: mobile dental units.
2. Counties with low dentist coverage rates in rural areas (e.g., Botoșani, Vaslui, Vrancea) may require targeted workforce redistribution strategies: financial and fiscal incentives for dentists.
3. Raising contract reimbursement caps could help attract more dentists to work under contract with NHIH.
4. The focus of public policies should be on programs that are aligned with the community’s needs. These policies should ensure equitable access to services, while also considering financial resources and the long-term stability of the system. In Romania, a key step in this direction is the development of oral health policies oriented toward disease prevention. They may be oriented toward developing community-level programs that increase awareness of the importance of oral health and its determinants, including education, motivation, awareness-raising, and the integration of oral health education into the school curriculum.
5. Access driven by curative treatment needs or medical emergencies differs substantially from regular or preventive access. Therefore, for long-term results, a curriculum that includes tracking behavioral outcomes, not just educational content, is recommended. Thus, sustainable, long-term strategies can be developed.

As mentioned in previous studies, access to dental services depends on a range of factors that shape health status, including parents’ education [23,24], preventive behavior, and oral health status [36,37,38,39,40]. These interconnected determinants need to be explored further in future research to enable the development of an effective and comprehensive oral health public policy.

The burden of oral diseases has changed very little over the last three decades, highlighting the need for new, more effective approaches to improve oral and general health. Many countries face a dual challenge. First, addressing oral health care unmet needs and second the prevention of other oral conditions [41].

It is shown in the existing evidence that the accessibility of the oral health system plays a key role in the way children’s oral health is addressed. This underlines the need for better integrated approaches. The main focus should be on the prevention of oral diseases. In addition, education-focused intervention with a tailored message to the target group should be implemented. Efforts to prevent oral disease and promote health can be strengthened through affordable and accessible services and effective public health initiatives. These measures can play an essential role in reducing social inequalities and improving oral health outcomes across the population [14,16].

Strengths: To our knowledge, this is the first study conducted in Romania that focuses on examining children’s oral health and the access to dental services from three perspectives: availability, self-reported service utilization, and assessment of normative health status.

Research Limit: Our study compiles data from the routine information system, with desk research and health examination of a sample of 3843 children. Our results show contextual facts, which allow only hypotheses related to the connection between the availability of services, their utilization, and the status of children’s oral health, but we do not provide proof of causality. Given the lack of disaggregated coverage data, the analyses were limited to an exploratory approach. We included oral health indicators and services use measured by survey, and services coverage calculated as no of dentists per 100,000 inhabitants. No other disaggregations are possible. The GDP/capita and educational level are not included.

These analyses are descriptive and exploratory, and no causal inferences can be drawn regarding the relationship between dentist availability, service utilization, and oral health outcomes. Emphasizing that the findings generate hypotheses for future analytical or longitudinal studies.

The implication of this research for the field of knowledge: understanding the barriers in schoolchildren’s access to oral health services could allow the definition of effective policies that ensure an equitable access to oral health services for all Romanian children, thus contributing to ensuring healthy lives and promoting well-being for all at all ages, according to the SDGs Agenda for 2030.

## 5. Conclusions

Even though legislative provisions state that children’s access to dental services is free, the Romanian health care system’s capacity to deliver these services is limited. To address barriers beyond formal access and availability to the dental care system (such as organizational constraints, uneven geographic distribution of dental services, limited preventive orientation of care from practitioners or parents of children, or socio-cultural factors influencing dental care-seeking behavior), targeted interventions are needed. Key areas requiring improvement to reduce inequalities in access include the territorial distribution of dentists, the level of funding allocated to dental services by the NHIH, and the level of awareness regarding the importance of oral health. Public policies should be adopted to ensure that all children have access to at least one dental check-up per year through schools, public and private services, and educational programs targeting children and parents.

## Figures and Tables

**Table 1:** Spearman’s correlations within the four caries indices, the pattern of dental visits, and dentist coverage rate.

			Mean_dmfs	Mean_dmft	Mean_DMFS	Mean_DMFT	% Schoolchildren with Fewer than Two Dental Visits	Dentist Coverage Rate
Spearman’s rho	Mean_dmfs	Correlation Coefficient	1.000	0.775 **	0.565 **	0.433 **	0.007	−0.162
		Sig. (2-tailed)	.	<0.001	<0.001	0.008	0.967	0.344
		N	36	36	36	36	36	36
	Mean_dmft	Correlation Coefficient	0.775 **	1.000	0.489 **	0.594 **	−0.084	−0.036
		Sig. (2-tailed)	<0.001	.	0.002	<0.001	0.627	0.837
		N	36	36	36	36	36	36
	Mean_DMFS	Correlation Coefficient	0.565 **	0.489 **	1.000	0.902 **	−0.186	−0.067
		Sig. (2-tailed)	<0.001	0.002	.	<0.001	0.278	0.696
		N	36	36	36	36	36	36
	Mean_DMFT	Correlation Coefficient	0.433 **	0.594 **	0.902 **	1.000	−0.232	0.031
		Sig. (2-tailed)	0.008	<0.001	<0.001	.	0.173	0.856
		N	36	36	36	36	36	36
	% schoolchildren with fewer than two dental visits	Correlation Coefficient	0.007	−0.084	−0.186	−0.232	1.000	−0.172
		Sig. (2-tailed)	0.967	0.627	0.278	0.173	.	0.316
		N	36	36	36	36	36	36
	Dentist coverage rate	Correlation Coefficient	−0.162	−0.036	−0.067	0.031	−0.172	1.000
		Sig. (2-tailed)	0.344	0.837	0.696	0.856	0.316	.
		N	36	36	36	36	36	36

** Correlation is significant at the 0.01 level (2-tailed).

## Data Availability

The data presented in this study are available on request from the corresponding author upon reasonable request due to ethical and legislative reasons.

## Abbreviations

The following abbreviations are used in this manuscript:

SDG	Sustainable Development Goals
NHIH	National Health Insurance House
DMFT/S	Decayed, Missing, Filled Permanent Teeth or Surfaces
dmft/s	Decayed, Missing, Filled Primary Teeth or Surfaces
GDP	Gross Domestic Product
